# Arthroscopic versus open cancellous bone grafting for scaphoid delayed/nonunion in adults (SCOPE-OUT): study protocol for a randomized clinical trial

**DOI:** 10.1186/s13063-023-07281-5

**Published:** 2023-04-14

**Authors:** Morten Kjaer, Jeppe Vejlgaard Rasmussen, Robert Gvozdenovic

**Affiliations:** 1 Gentofte Hospitalsvej 1, Opg. 17 St, 2900 Hellerup, Denmark; 2 Department of Orthopedic Surgery, Hand Surgery Unit, Copenhagen University Hospital Herlev/Gentofte, 2900 Hellerup, Denmark; 3 Department of Orthopedic Surgery, Shoulder and Elbow Surgery Unit, Copenhagen University Hospital Herlev/Gentofte, 2900 Hellerup, Denmark

**Keywords:** Scaphoid, Scaphoid nonunion, Cancellous, Graft, Arthroscopic, Time to union, Randomized controlled trial

## Abstract

**Background:**

Scaphoid non-union results in pain and decreased hand function. Untreated, almost all cases develop degenerative changes. Despite advances in surgical techniques, the treatment is challenging and often results in a long period with a supportive bandage until the union is established. Open, corticocancellous (CC) or cancellous (C) graft reconstruction and internal fixation are often preferred. Arthroscopic assisted reconstruction with C chips and internal fixation provides minimal trauma to the ligament structures, joint capsule, and extrinsic vascularization with similar union rates. Correction of deformity after operative treatment is debated with some studies favouring CC, and others found no difference. No studies have compared time to union and functional outcomes in arthroscopic vs. open C graft reconstruction. We hypothesize that arthroscopic assisted C chips graft reconstruction of scaphoid delayed/non-union provides faster time to union, by at least a mean 3 weeks difference.

**Methods:**

Single site, prospective, observer-blinded randomized controlled trial. Eighty-eight patients aged 18–68 years with scaphoid delayed/non-union will be randomized, 1:1, to either open iliac crest C graft reconstruction or arthroscopic assisted distal radius C chips graft reconstruction. Patients are stratified for smoking habits, proximal pole involvement and displacement of > / < 2 mm. The primary outcome is time to union, measured with repeated CT scans at 2-week intervals from 6 to 16 weeks postoperatively. Secondary outcomes are Quick Disabilities of the Arm, Shoulder and Hand (Q-DASH), visual analogue scale (VAS), donor site morbidity, union rate, restoration of scaphoid deformity, range of motion, key-pinch, grip strength, EQ5D-5L, patient satisfaction, complications and revision surgery.

**Discussion:**

The results of this study will contribute to the treatment algorithm of scaphoid delayed/non-union and assist hand surgeons and patients in making treatment decisions. Eventually, improving time to union will benefit patients in earlier return to normal daily activity and reduce society costs by shortening sick leave.

**Trial registration:**

ClinicalTrials.gov NCT05574582. Date first registered: September 30, 2022. Items from the WHO trial registry are found within the protocol.

**Supplementary Information:**

The online version contains supplementary material available at 10.1186/s13063-023-07281-5.

## Background

A scaphoid fracture is the most common injury to the carpal bones. The incidence is 107–151/100.000 per year and fractures are predominantly sustained by males in their twenties [1]. Scaphoid non-union is defined as a lack of healing 6 months after injury and develops in 5–25% of cases after non-operative treatment [2, 3]. Delayed union is defined as incomplete healing 2–6 months after injury. However, some potential for a union probably exists, especially in nondisplaced fractures, otherwise this condition is associated with a transition into a persistent non-union [4]. The risk of non-union increases with delayed diagnosis and treatment, displaced fractures, proximal pole fractures, smoking, poor vascularity and advancing age [5–8].

The scaphoid is primarily covered with cartilage and has a retrograde blood supply. The dorsal branch of the radial artery accounts for 80% and a separate volar branch for 20% of the extramedullary blood supply. The proximal pole is only supplied by the intramedullary flow. Compromised blood supply can explain the potential of non-union and avascular necrosis of the proximal pole. The healing process can be complicated by volar angulation of the fracture leading to humpback deformity. This will disrupt carpal kinematics and result in lunate instability and dorsal intercalated segment instability (DISI). Untreated scaphoid non-union can lead to degenerative changes, called scaphoid non-union advanced collapse (SNAC), and irreversible impairment such as pain and altered hand function.

**Fig. 1:**
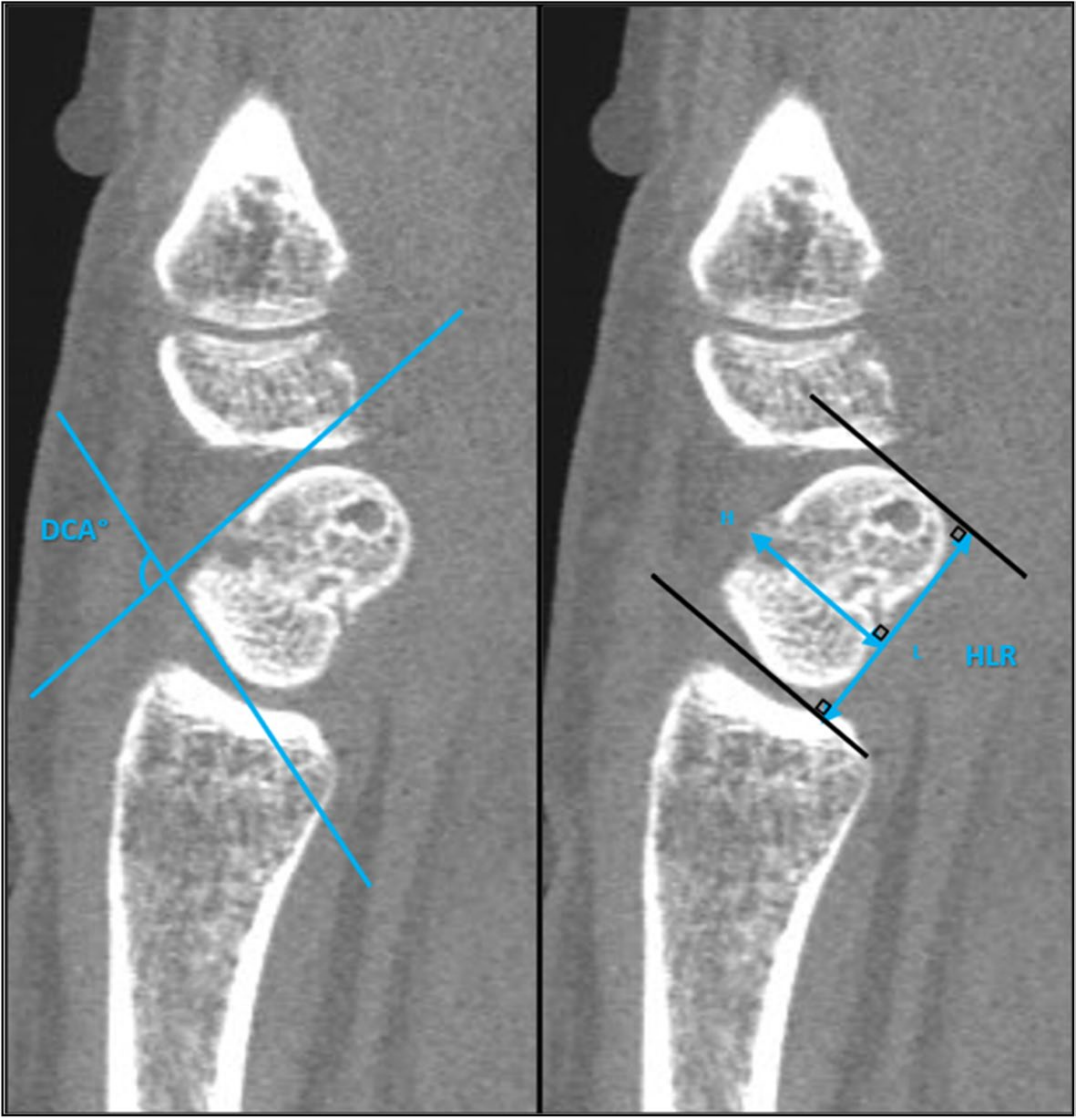
Sagittal CT scan of scaphoid non-union. The measurement of dorsal cortical angle (DCA) and height-length ratio (HLR). The height-length ratio is calculated by dividing the scaphoid height by length

X-ray is commonly applied to evaluate the scaphoid, although CT scans are reportedly superior in terms of displacement, angulation and union [9]. Different measurements to describe the angulation and deformity of the scaphoid are suggested. The height length ratio (HLR) and dorsal cortical angle (DCA) (Fig. 1) are found to be the most reliable measurements [10, 11].

Surgical treatment of scaphoid delayed/non-union is technically demanding and often results in a long period with a supportive bandage until union is established. Current treatment strategies for delayed union and non-union include vascularized or non-vascularized bone graft with internal fixation. Kirchner wires or screws have been the gold standard for fixation. A meta-analysis found an average of 94% union with screw fixation and 77% with K-wire fixation in unstable non-union [12]. Open CC graft reconstruction is often preferred [13, 14]. However, there is an increased donor site morbidity from the iliac crest, and the outcome is not superior to distal radius C graft [15]. Arthroscopic reconstruction with C chips and internal fixation is predominantly applied in delayed union and stable non-union [16–18]. The advantages of arthroscopy include thorough wrist assessment, evaluation of concomitant ligamentous injury and minimal trauma to the ligament structures, joint capsule and the tenuous blood supply [19]. Recent studies have reported an 86–100% union rate, also in patients with cystic changes and/or translation > 2 mm, and/or humpback deformity [20–24]. A retrospective study, with 62 cases, found union in 96% arthroscopic group vs. 97% in open surgery with no difference in the functional results, however, time to union was not recorded [16]. No RCT comparing arthroscopic or conventional open C graft technique have been performed.

The objective of this study protocol is to describe the methodology for a randomized controlled trial comparing time to union and functional outcome scores of arthroscopic assisted C chips reconstruction or open C graft reconstruction for scaphoid delayed/non-union. The standard protocol items recommendations for interventional trials (SPIRIT) statement 2013 have been followed for the completion of this protocol (Additional file 1) and the items from the WHO trial registry are found in within the protocol.

### Hypothesis

Arthroscopic assisted C chips graft reconstruction of scaphoid delayed/non-union is superior to open C graft reconstruction regarding faster time to union, by at least a mean 3 weeks difference.

## Method and analyses

### Study design

This is a single-centre, 1:1 observer-blinded randomized controlled, superiority trial. The main objective is to compare open C graft with arthroscopic assisted C chips graft reconstruction for scaphoid fractures with delayed/non-union.

## Subjects

A total of 88 patients with scaphoid delayed/non-union are randomized to either:

1. Group A – Arthroscopic assisted C chips graft reconstruction (intervention group), *n* = 44
2. Group O – Open C graft reconstruction (control group), *n* = 44

## Inclusion criteria

1. Patients aged 18–68 years.
2. A scaphoid fracture without healing 3–6 months since fracture (delayed union) for cases with either displacement > 1 mm or comminution and failed non-operative treatment.
3. Scaphoid fracture without healing > 6 months since fracture (non-union) regardless of displacement, comminution and if previous non-operative treatment has been tried.
4. ASA 1–3.

## Exclusion criteria

1. Open fractures
2. Associated trans-scaphoid perilunate dislocation.
3. Associated fracture in the hand/upper extremity.
4. Previous failed surgical treatment for scaphoid delayed/non-union.
5. Stage 2 SNAC or above.
6. Avascular necrosis of the proximal pole as evaluated with MRI and absence of punctate bleeding intraoperatively.
7. Patients with gross humpback deformity of HLR > 0.75 and/or DCA < 70°.
8. Patients unable to understand instructions in Danish, complete the rehabilitation protocol, or answering the questionnaires because of physical or cognitive impairment, as evaluated by the surgeon at the first visit.

## Enrolment

**Fig. 2:**
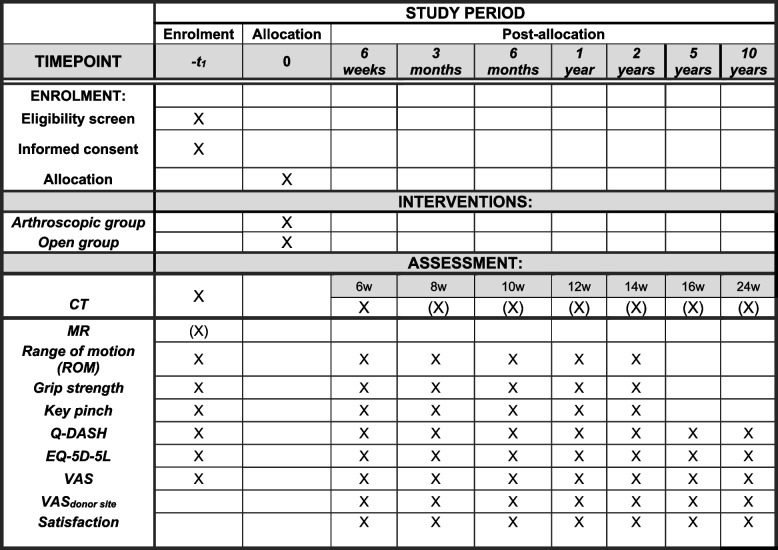
Schedule of study enrollment and assessment

All Danish citizens aged 18–68 years with scaphoid delayed/non-union referred to the hand surgy unit, Herlev and Gentofte University Hospital, will be offered participation in the study. Other hand unit departments in the Capital Region of Denmark will forward their referrals to our department for inclusion. The physician will review the medical records and assess whether the patients fulfil the inclusion/exclusion criteria. If the patient is found eligible, they will receive oral and written information of the study (Fig. 2) and the possibility to bring a bystander for the preparation consultation. The patients will be given a minimum of 24 h deliberation period. At the preparation consultation with the primary investigator, the patient will once again receive oral and written information, and trial risks of the study, and subsequently the consent will be obtained. The patients will simultaneously be allocated to one of the treatment groups. Patients can withdraw their consent at any time. The Helsinki Declaration will be followed [25]. The informed consent will give the primary investigator access to information about sex, age, comorbidity, trauma, time from fracture to treatment, history of smoking, occupation, hand dominance, pain, ongoing workers’ compensation and insurance claim. Baseline range of motion, grip strength, Key-pinch strength, pain in rest and activity (VAS) and Q-DASH will be recorded (Fig. 2).

Patients will undergo a clinical examination and CT scan of the wrist to describe angulation (HLR and DCA), displacement, localization of non-union, presence of cysts and degenerative changes. All patients with involvement of the proximal pole will undergo gadolinium-enhanced MRI to clarify vascularity, but the final assessment of avascular necrosis of the proximal pole will be performed perioperatively. If punctate bleeding after the tourniquet being off for at least 5 min of expectation cannot be visible, the patient will then be excluded from the study and will be simultaneously operated on with a vascular bone graft or a salvage procedure.

The operations will be performed at the Orthopedic Department at Herlev and Gentofte University-Hospital, Denmark, which is the largest referral hand unit in Denmark. Annually 1–25 patients are treated for scaphoid delayed/non-union. With forwarded referrals included, we estimate a 3-year inclusion period from 01–01-2023 to 01–01-2026.

## Randomization

Based on the sample size calculation, a total number of 88 patients will be allocated into two groups of equal size.

1. Group A – Arthroscopic assisted C chips graft reconstruction (intervention group), *N* = 44.
2. Group O – Open C graft reconstruction (control group), *N* = 44.

The randomization is done in the outpatient clinic and the patients will be informed about the operative treatment. The randomization is done using an irreversible application in Research Electronic Data Capture (REDCap). Patients will be allocated in a 1:1 ratio by block randomization, stratified for proximal pole fracture (yes /no), dislocation (> / < 2 mm) and smoking (yes/ no). An independent statistician generated the randomization sequence for REDCap, and thus the allocation is concealed to the research staff and physicians.

## Blinding

In this observer-blinded RCT, union is assessed by a blinded musculoskeletal radiologist. QDASH is a patient-reported survey, without the involvement of surgeons or research staff. Other secondary outcomes will be measured by an independent observer. The study will not be blinded to the operating theatre staff, surgeons, physiotherapists or patients.

## Interventions

All patients will be operated on by 4 highly experienced hand surgeons of the same experience level. Two surgeons will perform the open technique and 2 surgeons the arthroscopic technique. The surgeons are using the technique which they are familiar with to avoid a learning curve affecting the outcomes.

## Group A: arthroscopic assisted cancellous chips graft reconstruction

Patients are awake in a supine position with the arm anaesthetized as standard. The arm is attached to a wrist traction tower with vertical traction of 5–8 kg force through plastic finger trap devices. An arm tourniquet is applied. Through 3/4 and 4/5 portals using dry-arthroscopy, the radio-carpal joint is inspected for cartilage damage, synovitis and ligament injuries. Mid-carpal radial (MCR) and mid-carpal ulnar (MCU) portals are applied to examine the non-union site. The non-union site is debrided with a shaver until healthy-looking bone with punctate bleeding. The C chips graft is harvested from the ipsilateral distal radius through a 2 cm incision with a biopsy needle, using multiple biopsy probes. After arthroscopy, the wrist is taken out of traction and the non-union is reduced under the C-arm image intensifier and a 1.2-mm K-wire is inserted percutaneously, either in a retrograde or anterograde manner. The scaphoid is prepared with a drill, the length is measured and the graft is inserted with a trochar through the MCR portal. An Acutrak mini compression screw (Acumed^tm^, Hillsboro, USA) is inserted. The final position is confirmed under fluoroscopy. The non-union site is inspected, and spilled bone graft material is impacted in the gap. Finally, the skin is sutured, and a bandage is applied.

## Group O: open corticocancellous graft reconstruction

Patients are under general anaesthesia with the arm anaesthetized with a regional block anaesthesia. A longitudinal volar incision, lateral to the flexor carpi radialis (FCR) curved distal over the scaphoid tuberosity is made. The FCR is retracted to the ulnar side and the volar capsule and ligaments are incised longitudinally to expose the scaphoid. The non-union is debrided to a healthy-appearing bone. A C graft is harvested from the ipsilateral iliac crest through a 4-cm incision. The cancellous graft is prepared and placed in the cavity. Under fluoroscopy, a 1.2-mm K-wire is inserted in a retrograde or anterograde manner. After drilling, the length is measured and an Acutrak mini compression screw (Acumed^tm^, Hillsboro, USA) is inserted. The final position is confirmed under fluoroscopy. The capsule and ligaments are repaired, the skin is sutured and the immobilizing bandage is applied.

## Physiotherapy and rehabilitation

Rehabilitation will be identical between the groups. Stitches are removed 2 weeks postoperatively. Patients will be provided a thumb/wrist splint for 2 weeks, followed by the application of removable orthosis for another 4 weeks allowing the beginning of light non-weight-bearing exercises. The wrist will be allowed mobilization without restrictions if union is established on a postoperative CT scan and with the absence of scaphoid tenderness on the clinical examination. Return to work will be accepted when the union is established.

### Outcome measures

#### Primary outcome

Time to union will be assessed with repeated CT scans in 2 weeks intervals from 6 to 16 weeks postoperative. If union is not achieved within 16 weeks, a CT scan will be made 24 weeks postoperatively. If union is not achieved at that point, the patient will be presented for another treatment modality. Union will be proclaimed and recorded when > 50% bone bridging occurs on CT scan [26, 27]. The Minimal clinically important differences (MCID) have not been defined. We use an arbitrary value of 3 weeks difference.

#### Primary functional outcome

*The Quick Disability of the Arm, Shoulder, and Hand* (Q-DASH) is a patient-reported survey [28, 29]. It is a subset of 11 items from the 30-item DASH questions that assess difficulties with specific tasks: 5 concerning symptoms, 4 on social function and 1 on work function, sleep and confidence. The score ranges from 0 to 100 and the higher score reflects disabilities. The MCID has been defined as 10.8 (range, 5–15) for comparable patients [30].

#### Secondary outcomes

For *radiographic outcomes*, the union rate will be evaluated with CT scans undertaken every second week. DCA and HLR will be measured preoperative and at follow-up on CT scans to evaluate the correction of deformity [11, 31].

*Pain* at rest and activity is recorded on a visual analogue scale (VAS), ranging from 0 to 10, with 10 reflecting the worst and 0 representing no pain in the wrist.

*Donor site morbidity* located to the iliac crest (Group O) and distal radius (Group A) are evaluated with VAS ranging from 0 to 10, with 10 reflecting the worst and 0 representing no pain in the wrist.

*Range of motion* (ROM) is measured in degrees with a goniometer and recorded in arcs for flexion/extension, supination/pronation and radial/ulnar deviation, compared to the unaffected wrist.

*Grip strength* is measured in kilograms with a Jamar dynamometer with the elbow will be in 90° flexion and attached to the chest compared to the unaffected [32].

*Key pinch* is measured in kilograms using a pinch gauge with the elbow in 90° flexion and attached to the chest, compared to the unaffected wrist [33].

*Patient satisfaction* is evaluated with the following question: What is the function of your hand today, compared to before surgery? With the following answer options: (1) disaster, (2) much worse, (3) slightly worse, (4) unchanged, (5) slightly better, (6) much better, (7) recovered. Second, for future research perspective, patients are asked: when you consider the following parameters: the activities you can carry out in daily life, your pain, your function of the hand, do you think your current situation is satisfactory? (yes/no) [34].

*EQ5D-5L* will be used to estimate the threshold for acceptable cost-utility ratio — the threshold for how much healthcare providers will pay for an extra quality-adjusted life year (QALY). The cost-utility of the arthroscopic and open techniques will be compared. Baseline and 2-year follow-up scores will be compared. A cost model will be defined from patient data, clinical records, and unit costs from the Danish healthcare system. Length of hospital stay, discharge, pain medication usage and readmission will be recorded.

## Complications and secondary surgery

We will record all complications related to the operative treatment (tendon, ligament, nerve or arterial injury, infection, complex regional pain syndrome, haematoma or hardware failure). Reoperations, defined as revision surgery and secondary surgery due to no union will be noted.

## Follow-up

All patients will be followed for 10 years. Union will be measured in 2 weeks intervals 6–16 weeks after operative treatment. Clinical outcomes and patient-reported outcomes will be measured after 1.5, 3, 6, 12 and 24 months. Online questionnaires will be sent after 5 and 10 years (Fig. 2).

## Protocol violations and patient drop-out

**Fig. 3:**
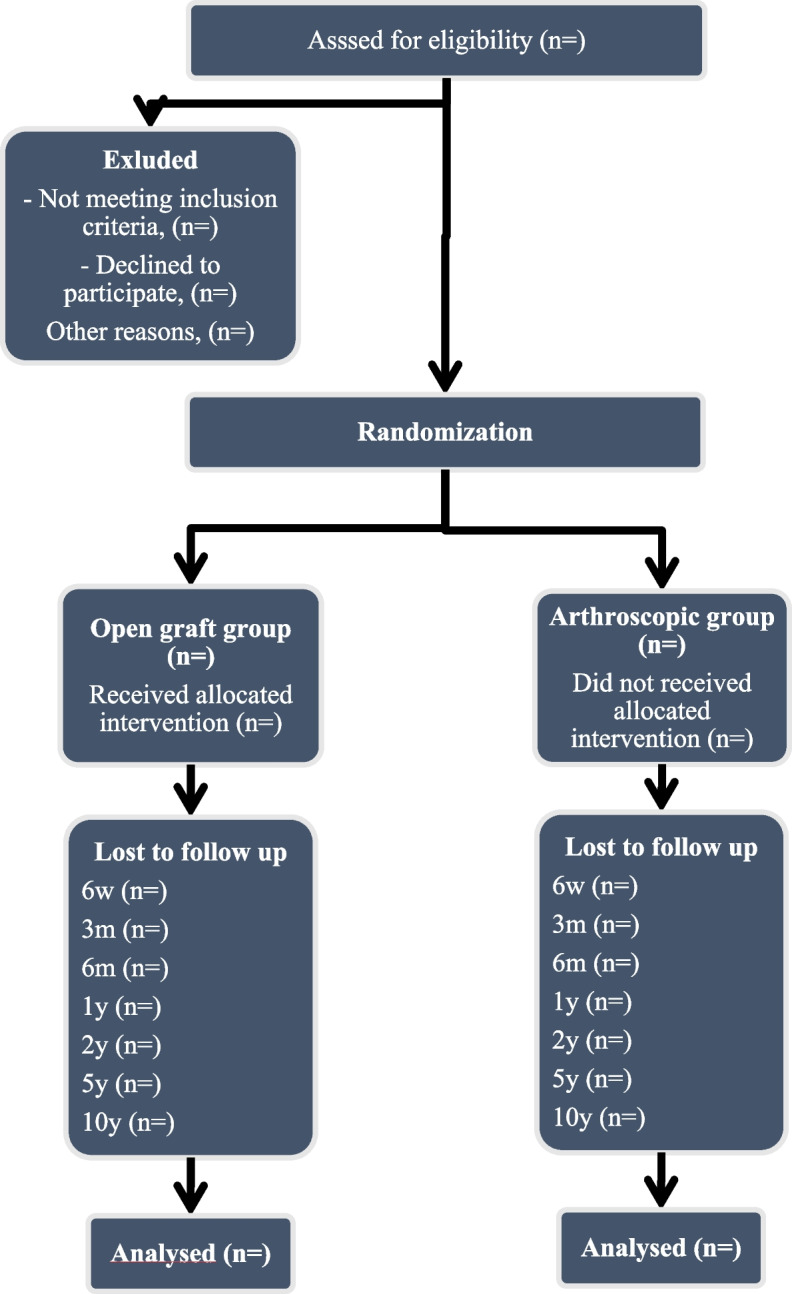
Consort flow diagram. Flow of patient through the study

The patients evaluated for inclusion will be reported in a consort diagram (Fig. 3). The statistical analysis will be conducted on an intention-to-treat basis. Outcome scores will be analysed according to the initial study group assignment regardless of possible cross-over. If crossover does occur, a secondary per-protocol analysis will be conducted.

Patients drop-out/loss to follow-up will be recorded and the reason will be noted. The patient is included in the analysis with their latest follow-up if at least a 6-week evaluation is available. Patients who undergo revision/secondary surgery will remain in the study and will be followed according to the index procedure. Their results before and after the revision procedure are included in the analysis. If challenges in the trial conduct occur, they will be presented by the primary investigator every second week to the research group. Any protocol modifications will be communicated to all investigators, observers and trial registries immediately. No regular audit is planned, apart from possible independent inspection visits by the Data Protection Agency and the National Committee on Health Research Ethics.

## Adverse events

The operative techniques are used routinely in our department and are considered safe, therefore interim analysis will not be conducted. At follow-up, patients will be asked about complications, and journal notes about hospital contacts related to the surgical procedure are recorded.

### Statistics

#### Sample size calculation

The sample size calculation was performed with an expected mean time to union of 56 days and a SD of 30 days in Group A and 77 days and SD on 40 days in group B. The sample size required in each group to provide 80% power to detect a mean 3 weeks difference [13], with two-sided alpha on 0.05, was 36 patients in each group. Considering dropouts and loss to follow-up with a rate of 20% the final number of patients needed in total for this study is 88 patients.

#### Data analysis

Interim data analysis will not be carried out. Continuous data will be presented as means with SD or as median with interquartile range (IQR) depending on the nature of data. Categorical data will be presented as counts with percentages. Differences in demographic data and outcome measures between groups will be compared using chi-square for categorical data and Student’s *t*-test (parametric data) or Wilcoxon signed rank test (non-parametric data) for continuous data.

An intention-to-treat analysis will be conducted for the primary outcome. A per-protocol analysis will be conducted to test the non-adherence to the protocol, and patients who do not comply with the protocol will be excluded. Primary outcome will be analysed with a mixed effect model adjusted for fracture location, smoking habits and dislocation (the strata in the block randomization) to account for imbalance in baseline covariates and to improve the power of the treatment effect.

Subgroup analyses for the duration of non-union (< 6 months vs. > 6 months), patients with/without deformity based on the preoperative measurements of HLR and DCA will be performed. It will be clarified if post hoc analyses are carried out. For missing data in an outcome measure, multiple imputation analysis with the covariates: age, smoking, fracture location, dislocation > / < mm and duration of non-union will be conducted to minimize bias of nonresponse. Applied level of significance for all statistical analyses is *P* < 0.05. The statistical analyses will be performed using computerized statistical software.

#### Data management

Data will be stored securely at the study location. Data will be collected in REDCap electronic data capture tools and EPIC hospital clinical digital journal system hosted in the Capital Region of Denmark. REDCap is a secure web-based data collection application. To limit typos the applications is coded to independently audit the data and warn the investigator immediately in case of double data entry, values beyond normal ranges and/ or missing data values. During data entry, the application will remind the assessor to cross-check that the questionaries for the visit are completed and that the next visit is planned. If data for each patient is not entered in Redcap within 3 days of the expected visits, the application will send a reminder by mail to the primary investigator thus minimizing missing values in the dataset. Identifying information about the patients will be stored in secured hospital servers. The primary investigator will have access to the trial data and final dataset. The study is approved and monitored by the Danish Data Protection Agency (Pactius) and it will follow the General Data Protection Regulation and Data Protection Act. The anonymized results will be made available for meta-analysis and systematic reviews.

## Discussion

Today, operative treatment for scaphoid delayed union or non-union is performed with the conventional open technique or the arthroscopic technique based on the preference of the surgeon. Based on non-randomized studies both techniques appear safe and with no major differences in functional outcome or adverse events [16, 35]. There is, however, disagreement regarding the treatment of patients with a large deformity. Hegazy et al. [36] and Sayegh and Strauch [13] found superior correction and superior ROM, Q-DASH and Mayo wrist scores in patients treated with a structural graft compared with patients treated with a non-structural graft. Other studies found no difference in deformity correction [15, 37, 38].

The arthroscopic technique is potentially less invasive with minimal donor site morbidity and potentially faster time to union [15] because of minimal trauma to the ligament structures, joint capsule and the tenuous blood supply [19]. It may also have advantageous osteogenic properties compared to a structural graft [39]. Differences in time to union between the open technique and the arthroscopic technique have, to our knowledge, never been investigated.

X-ray is commonly applied to monitor union after operative treatment for scaphoid delayed/non-union. Consolidation on at least three out of four views is a sign of union [40]. X-ray is not reliable to determine fracture displacement and deformity [41], and the wrong position of the hand and “overlining” in the fracture line can complicate the evaluation of union. CT scans are reportedly more accurate and reliable in the assessment of union [26, 42–44]. We aim to provide a more accurate measurement of time to union with repeated CT scans.

The study differentiates from standard treatment with up to 7 scheduled CT scans in patients with prolonged union time, compared to 1–3 CT scans and 1–3 X-rays as a standard follow-up. The background radiation in Denmark is 3 mSv each year. Each CT scan is performed with the involved wrist above the head with a mean radiation dose of 0.03 mSv. The wrist contains minimal radiation-sensitive red bone marrow and the cumulative radiation dose is maximal 0.21 mSv, (category IIA, international commission on radiation Protection), corresponding to 21 days of background radiation [45]. The radiation dose of each scaphoid X-ray is 0.0002 mSv.

We aim to decrease observer and selection bias through blinding and randomization, stratified for tobacco-smoking habits, fracture displacement (> / < 2 mm) and proximal pole fractures. By adding 20% to the sample size, we compensate for dropouts and attempt to avoid Type-II errors. Potential dropouts will be minimized with permission to contact patients by mail or phone if they do not show up for follow up-visit or if data is missing.

The patients will not be blinded to their treatment allocation. Sham surgery can be used to blind patients regarding skin incisions and subsequently the surgical technique. However, all patients treated with open bone grafting using a cancellous graft taken from the iliac crest will be operated on in general anaesthesia, while arthroscopically treated patients undergoing the treatment in regional block anaesthesia. Furthermore, the use of graft from the iliac crest in one group and not the other makes it difficult to blind patients as donor-side pain is expected. Finally, we do not expect patients to have an expectation of open- or arthroscopic-assisted graft reconstruction to be superior to the other.

Observer bias is minimized with a blinded musculoskeletal radiologist for the primary outcome, to prevent observer bias from favouring one of the interventions.

The secondary outcome (Q-DASH) is reported by the patient independently, thus with no implication to the assessor. Other secondary outcomes are evaluated by an independent, non-blinded observer.

The current standard treatment at our department includes follow-up until the union is established. In this study, we will have additional follow-up examinations at 6 months and at 1 and 2 years. It has the risk of keeping the patients in the role of being ill. However, the patients can have a feeling of extra good care with the possibility to address uncertainty or problems more easily.

Patients are at risk of being treated inferior with an intervention which is deemed inferior with the study analysis, but nothing a priori suggests which intervention is the better one.

The trial will provide high-quality evidence regarding time to union and short- and long-term functional outcomes of open and arthroscopic assisted graft reconstruction for scaphoid delayed/non-union.

The results from the study can contribute to establish a treatment algorithm for scaphoid delayed/non-union together with results from other studies.

### Trial status

Version 2.0, February 3, 2023.

Start of inclusion: February 15, 2023.

Finish date of recruitment: February 15, 2026

## Supplementary Information

**Additional file 1.** Spirit Checklist

**Additional file 2.** Translation Ethical approval

**Additional file 3.** Approval, National Committee on Health Research Ethics

**Additional file 4.** Funding document, English translation

**Additional file 5.** Funding document, original Danish version

## Data Availability

The electronic data will be stored in closed drivers and on REDCap. Printed data will be stored in closed cabinets. The deidentified dataset and results will be available from the primary investigator on request.

## Abbreviations

RCT: Randomized controlled trial
CC: Corticocancellous
C: Cancellous
C-chips: Cancellous chips
CT: Computed tomography
AVN: Avascular necrosis
Q-DASH: Quick Disability of the Shoulder and Hand
VAS: Visual analogue scale
QALY: Quality-adjusted life year
ROM: Range of motion
MRI: Magnetic resonance imaging
SNAC: Scaphoid non-union advanced collapse
DISI: Dorsal intercalated segment instability
DSA: Dorsal cortical angle
HLR: Height-length ratio
MCR: Mid-carpal radial
MCU: Mid-carpal ulnar
FCR: Flexor carpi radialis
MCID: Minimal Clinical Important Difference
SD: Standard deviation
IQR: Interquartile range
REDCap: Research Electronic Data Capture
