# Supplementary material for: Arthroscopic versus open cancellous bone grafting for scaphoid delayed/nonunion in adults (SCOPE-OUT): study protocol for a randomized clinical trial
Source: Trials. 2023 Apr 14;24:273. doi: 10.1186/s13063-023-07281-5 (PMC10103438; doi:10.1186/s13063-023-07281-5)
Supplement: Supplementary file 2 — Additional file 2. Translation Ethical approval [file 13063_2023_7281_MOESM2_ESM.pdf]

Center for Regional Udvikling

De Videnskabsetiske Komitéer
Kongens Vænge 2
3400 Hillerød

Afsnit Enhed for Sundhedsforskning og Innovation
Telefon 38666395
Direkte 38666395
Web www.regionh.dk

Journal-nr.: H-21075664

Dato: 08-07-2022

Morten Kjær
Herlev/Gentofte Hospital
Håndkirurgisk afdeling, afdeling for led og knoglesygdomme
Gentofte Hospitalsvej 1
2900 Hellerup

H-21075664 - Kikkertassisteret versus åben kirurgisk rekonstruktion med knogletransplantat i behandlingen af bådbenets manglende heling efter brud (scaphoideum pseudoartrose)

Endelig godkendelse.

Afgørelsen er truffet efter lovbekendtgørelse nr. 1338 af 1. september 2020 - lov om videnskabsetisk behandling af sundhedsvidenskabelige forskningsprojekter og sundhedsdatavidenskabelige forskningsprojekter.

Jeg bekræfter modtagelsen af mail af 8. juli 2022, som svar på afgørelse af 17. juni 2022, hvori der opstilledes betingelser for godkendelsen af projektet.

Betingelserne for godkendelsen anses for opfyldt. Projektet er dermed endeligt godkendt.

Godkendelsen gælder til den 1. juni 2022 og omfatter følgende dokumenter:

- Forsøgsprotokol, version 3.0, af 1. juli 2022
- Deltagerinformation, version 2.0, af 16. maj 2022
- Informeret samtykkeerklæring, version 1.0, af 6. december 2022

Godkendelsen gælder for de anmeldte forsøgssteder og den anmeldte forsøgsansvarlige i Danmark.

Komiteen er ikke ressortmyndighed for regelsættet om databeskyttelse. Komiteen forudsætter at projektet gennemføres i overensstemmelse med databeskyttelsesforordningen og databeskyttelsesloven.

Iværksættelse af projektet i strid med godkendelsen kan straffes med bøde eller fængsel, jf. komitélovens § 41.

Ændringer

Foretages der væsentlige ændringer i protokolmaterialet under gennemførelsen af projektet, skal disse anmeldes til komiteen i form af tillægsprotokoller. Ændringerne må først iværksættes efter godkendelse fra komiteen, jf. komitélovens § 27, stk. 1.

Anmeldelse af tillægsprotokoller skal ske elektronisk på www.drvk.dk/anmeldelse med det allerede tildelte anmeldelsesnummer og adgangskode.

Væsentlige ændringer er bl.a. ændringer, der kan få betydning for forsøgspersonernes sikkerhed, fortolkning af den videnskabelige dokumentation, som projektet bygger på samt gennemførelsen eller ledelsen af projektet. Det kan fx være ændringer i in- og eksklusionskriterier, forsøgsdesign, antal forsøgspersoner, forsøgsprocedurer, behandlingsvarighed, effektparametre, ændringer om de forsøgsansvarlige eller forsøgssteder samt indholdsmæssige ændringer i det skriftlige informationsmateriale til forsøgspersonerne.

Hvor nye oplysninger betyder, at forskeren overvejer at ændre proceduren eller stoppe forsøget, skal komiteen orienteres om det.

Bivirkninger og hændelser

Løbende indberetning

Komiteen skal omgående underrettes, hvis der under projektet optræder formodet alvorlige, uventede bivirkninger eller alvorlige hændelser, jf. komitélovens § 30, stk. 1. Indberetningen skal ledsages af kommentarer om eventuelle konsekvenser for forsøget. Det er kun bivirkninger og hændelser forekommet i Danmark, der skal indberettes. Underretning skal ske senest 7 dage efter, at sponsor eller den forsøgsansvarlige har fået kendskab til tilfældet.

Ved indberetning kan anvendes et skema, der findes på www.nvk.dk. Skemaet med bilag kan indsendes elektronisk ved anvendelse af digital signatur.

Årlig indberetning

Én gang årligt i hele forsøgsperioden skal komiteen have tilsendt en liste over alle formodet alvorlige (ventede og uventede) bivirkninger og alvorlige hændelser, som er indtruffet i forsøgsperioden sammen med en rapport om forsøgspersonernes sikkerhed, jf. komitélovens § 30, stk. 2.

Materialet skal være på dansk eller engelsk.

Ved indberetning skal anvendes et skema, der findes på www.nvk.dk. Skemaet med bilag kan indsendes elektronisk ved anvendelse af digital signatur.

Afslutning

Den forsøgsansvarlige og en evt. sponsor skal senest 90 dage efter afslutningen af projektet underrette komiteen herom, jf. komitélovens § 31, stk. 1. Projektet regnes som afsluttet, når sidste forsøgsperson er afsluttet.

Afbrydes projektet tidligere end planlagt, skal en begrundelse herfor sendes til komiteen senest 15 dage efter, at beslutningen er truffet, jf. komitélovens § 31, stk. 2.

Hvis projektet ikke påbegyndes, skal dette samt årsagen hertil meddeles komiteen.

Komiteen beder om kopi af den afsluttende forskningsrapport eller publikation, jf. komitelovens § 28, stk. 2. Vi skal i den forbindelse gøre opmærksom på, at der er pligt til at offentliggøre både negative, positive og inkonklusive forsøgsresultater, jf. komitélovens § 20, stk. 1, nr. 8.

Pligten til at indberette afsluttende forsøg og rapport påhviler forsøgsansvarlig og en evt. sponsor i forening.

Tilsyn

Komiteen fører tilsyn med, at projektet udføres i overensstemmelse med godkendelsen, jf. komitélovens § § 28 og 29.

Underskrift på samtykkeerklæringen

Komiteen gør opmærksom på, at forsøgsansvarlig kan delegere sin pligt til at underskrive samtykkeerklæringen til den person, der holder den mundtlige informationssamtale. Der skal i så fald være en skriftlig delegation hertil på forsøgssitet.

Databeskyttelse - fortegnelseskrav

Du skal være opmærksom på, at du kan være forpligtet til at få forskningsprojektet fortegnet.

Er du forsker ansat i Region Hovedstaden, gør du dette ved at rette henvendelse til Videnscenter for Dataanmeldelser i Region Hovedstaden, som er den regionale enhed, der administrerer forskningsfortegnelsen. Du kan læse mere om fortegnelsen og finde kontaktoplysninger på videnscenterets hjemmeside.

Er du ikke ansat i Region Hovedstaden, kan du orientere dig om fortegnelseskravet i Vejledning om fortegnelse på Datatilsynets hjemmeside.

Med venlig hilsen

Sofie Pohl
Studentermedhjælper
