# Supplementary material for: Arthroscopic versus open cancellous bone grafting for scaphoid delayed/nonunion in adults (SCOPE-OUT): study protocol for a randomized clinical trial
Source: Trials. 2023 Apr 14;24:273. doi: 10.1186/s13063-023-07281-5 (PMC10103438; doi:10.1186/s13063-023-07281-5)
Supplement: Supplementary file 3 — Additional file 3. Approval, National Committee on Health Research Ethics [file 13063_2023_7281_MOESM3_ESM.pdf]

Region Hovedstaden

Center for Sundhed

De Videnskabsetiske Komitéer
Borgervænget 3, st.
2100 København Ø

Morten Kjær
Sendt digitalt

Afsnit Sekretariatet
Telefon 3866 6395
Direkte 3866 6395
Web www.regionh.dk

Journal-nr.: H-21075664

Dato: 21-09-2022

H-21075664 - Kikkertassisteret versus åben kirurgisk rekonstruktion med knogletransplantat i behandlingen af bådbenets manglende heling efter brud (scaphoideum pseudoartrose)

The Committees on Health Research Ethics in the Capital Region of Denmark hereby confirm that above research project is approved and registered to be completed by 1st of June 2027.

Generally, an approved project is valid throughout the accepted period, and a regular confirmation of the approval does not take place. Unless otherwise stated, the approval includes all documents listed in the approval letter. Extension of a study might be approved when applied for later.

According to Danish law all health research, taking place in Denmark, must be approved by an Ethical Committee and must comply with Danish legislation. Please note, that "GCP" and "ICH-GCP" rules are only partially implemented in Danish law.

For additional inquiries, please contact the Secretariat for the Committees on Health Research Ethics in the Capital Region at +45 3866 6395 or by e-mail vek@regionh.dk

Kind regards,

Dorthe Frisvad
Sekretær

The Secretariat for

The Committees on Health Research Ethics in the Capital Region of Denmark
