# Supplementary material for: Arthroscopic versus open cancellous bone grafting for scaphoid delayed/nonunion in adults (SCOPE-OUT): study protocol for a randomized clinical trial
Source: Trials. 2023 Apr 14;24:273. doi: 10.1186/s13063-023-07281-5 (PMC10103438; doi:10.1186/s13063-023-07281-5)
Supplement: Supplementary file 4 — Additional file 4. Funding document, English translation [file 13063_2023_7281_MOESM4_ESM.docx]

**Morten Kjaer
Department of orthopedic surgery**

**Subject: Award of Ph.D. scholarship from internal research funds**

Dear Morten Kjaer

It is a pleasure to inform you, that the research council at Herlev and Gentofte Hospital has assigned you for 6 months salary corresponding to a total of 289.885,08kr. From the internal research funds spring 2022 for the project in the department of orthopedic surgery:

**Scaphoid non-union: Treatment challenges and long-term consequences**

**How to use your fund resources**

1. Agree with your department management when the grant is to be used within a total of 6-month period of 24 months from today’s date.
2. Afterwards, request the payroll officer to enter your start and end date in staff-web on with the salary code ÅKUE. This award letter must be placed in the personnel matter. NOTE! This salary code must not be used for payment of the 6^th^ holiday week and extra shifts.
3. Concomitantly, the research unit must be informed of the start and end date on mail [HGH-FP-Forskning@regionh.dk](mailto:HGH-FP-Forskning@regionh.dk)

Please complete and submit the attached reporting sheet to the research unit no later than 1 month after the period has ended. Publications in the research topic must clearly indicate that they originate from Herlev and Gentofte University Hospital.

Kind regard on behalf of Research council Herlev and Gentofte

Bodil Ørkild

Chairman of the research council

cc: Department management
