# Supplementary material for: Arthroscopic versus open cancellous bone grafting for scaphoid delayed/nonunion in adults (SCOPE-OUT): study protocol for a randomized clinical trial
Source: Trials. 2023 Apr 14;24:273. doi: 10.1186/s13063-023-07281-5 (PMC10103438; doi:10.1186/s13063-023-07281-5)
Supplement: Supplementary file 5 — Additional file 5. Funding document, original Danish version [file 13063_2023_7281_MOESM5_ESM.pdf]

Herlev og Gentofte Hospital

Sekretariat og Kommunikation, Forskning

Kildegårdsvej 28, opg. 65
2900 Hellerup

Telefon +45 38 67 30 01
Mail HGH-FP-Forskning@Regionh.dk

Morten Kjær
Afd. for Led og Knoglekirurgi

Dato: 1. april 2022

Vedr.: Uddeling af ph.d.-stipendium fra Interne forskningsmidler

Kære Morten Kjær

Det er en glæde at kunne meddele dig, at Forskningsrådet på Herlev og Gentofte Hospital har tildelt dig 6 måneders løn svarende til i alt 289.885,08 kr. fra de Interne forskningsmidler forår 2022 til projektet i Afd. for Led og Knoglekirurgi:

Scaphoid non-union: Treatment challenges and long-term consequences

Sådan anvender du din tildeling

1. Aftal med din afdelingsledelse hvornår bevillingen skal bruges inden for en samlet 6 måneders periode på 24 måneder fra dags dato.
2. Herefter beder I afdelingens lønansvarlige om at indtaste din start- og slutdato i Personaleweb på med lønkoden ÅKUE. Dette tildelingsbrev lægges samtidig i personalesagen. NB! Denne lønkode må *ikke* bruges til udbetaling af 6. ferieuge og ekstra vagter.
3. Samtidig orienteres Forskningsenheden om start og slut på mail til HGH-FP-Forskning@regionh.dk

Du bedes udfylde og indsende vedlagte afrapporteringsskema til Forskningsrådet senest 1 måned efter perioden er slut. Publikationer om projektet skal tydeligt angive, at de udgår fra Herlev og Gentofte Universitetshospital.

Venlig hilsen på vegne af Forskningsråd Herlev og Gentofte

f Bodil Ørkild

Anne [illegible]

Formand for Forskningsrådet

cc: Afdelingsledelsen
